# Assessment of clinical trial participant patient satisfaction: a call to action

**DOI:** 10.1186/s13063-016-1616-6

**Published:** 2016-10-06

**Authors:** Bethann Mangel Pflugeisen, Stacie Rebar, Anne Reedy, Roslyn Pierce, Paul J. Amoroso

**Affiliations:** MultiCare Institute for Research and Innovation, 314 Martin Luther King Jr. Way, Suite 402, Tacoma, WA 98405 USA

## Abstract

**Background:**

As patient satisfaction scores become increasingly relevant in today’s health care market, we sought to evaluate satisfaction of the unique subset of patients enrolling in clinical trials in a research facility embedded within a community hospital system.

**Methods:**

We developed and deployed a patient satisfaction survey tailored to clinical trial patients who consented to and/or completed a clinical trial in our research institute in the prior year. The survey was distributed to 222 patients. Likert scale responses were analyzed using top box and percentile rank procedures. Correlation analysis was used to evaluate associations between the clinical trial experience and intent to return to our system for routine care.

**Results:**

Ninety surveys were returned in the 6 months following the mailing for a 41 % response rate; the bulk of these (*N* = 81) were returned within 6 weeks of the mailing. The questions with the highest ranking responses were related to interactions with staff (84th percentile or higher). Fifty-one point one percent of patients (64th percentile) strongly agreed that they would seek future care in our system. Patient intent to return to the provider seen during the clinical trial was most highly correlated with intent to seek future care within our system (*r* = 0.54, *p* < 0.0001). Reasons cited for clinical trial enrollment were generally altruistic.

**Conclusions:**

Querying this special patient population is feasible and yields valuable insight into their experience with healthcare system-based clinical trials and the relationship between clinical trial participation and perception of the healthcare system as a desirable resource for routine medical care. We argue that this work is invaluable to the research community and submit a call to action to our peers to begin systematic evaluation of clinical trial patient satisfaction.

**Electronic supplementary material:**

The online version of this article (doi:10.1186/s13063-016-1616-6) contains supplementary material, which is available to authorized users.

## Background

Patient satisfaction is an increasingly important measure in health care delivery. In the United States, commercial surveys, such as the Hospital Consumer Assessment of Healthcare Providers and Systems (HCAHPS) survey [1], are now conducted on a national scale and used routinely to satisfy internal and external demands for this information. In October 2012, the Centers for Medicare and Medicaid Services began to tie hospital reimbursements to standardized HCAHPS scores [2]. As the health care market becomes increasingly competitive and consumer-driven, patient satisfaction scores have the potential to influence consumer decisions about the utilization of health care resources, and studies have demonstrated positive associations between overall patient satisfaction and clinical outcomes [3, 4]. Standardization of patient satisfaction scores empowers health care consumers and leaders alike to evaluate and compare a consistent measure of the patient experience between institutions. Finally, it has been shown that patients who are “merely satisfied” with their health care experience are likely to seek future care elsewhere [5], an important finding for competing health care systems that are at risk of losing customers in response to low satisfaction.

Health care organizations with embedded research programs likely experience substantial crossover between research patients and patients seeking routine care; research may attract new patients to the system and the system can engage existing patients in research. Overall patient satisfaction has been shown to correlate most highly with communication with nurses [6], and research patients in an embedded research division interact with an additional team of study staff, often nurses and/or certified clinical research coordinators/assistants, who represent the larger organization. While the satisfaction of patients seeking routine care is now formally measured within most large health care systems [7], and the motivation of research patients for participating in clinical trials has been well-described [8–12], there is a dearth of information regarding the satisfaction of research patients with their clinical trial experience or the relationship between research patient satisfaction and use of the host institution for routine medical care. Notably, the 3rd International Clinical Trials Methodology Conference held in Glasgow in late 2015 included two posters addressing the patient experience of clinical trial participants. One systematic literature review (1995–2014) identified only five research patient experience measures [13], while a second reported results of a survey conducted with 44 clinical trial participants [14]. This suggests that while an international discussion about patient satisfaction is underway, it remains nascent.

Several unique aspects of the research patient experience, such as informed consent and patient incentives for study enrollment, are not part of routine care and thus cannot be evaluated by standardized patient experience surveys designed to assess the provision of nonresearch health care services. Two substantial gaps are created by the absence of a standardized and widely used survey explicitly designed to assess the unique patient experience of clinical trial participants: first, the research community operates in the absence of benchmarks by which to identify programmatic strengths/weaknesses and upon which to base improvements; second, the patient voice is lost.

We sought to develop an independent questionnaire that would (1) evaluate the satisfaction of patients participating in clinical trials within our research institute, (2) establish internal, measureable benchmarks to allow ongoing evaluation of service to our clinical trial participants, and (3) identify existing associations between patient engagement in clinical trials and use of our health care system for nonresearch medical care. Perhaps most importantly, however, this work is a call to action for the broader research community: it is time that we engage in a national/international conversation about the importance of measuring the experience of clinical trial patients and begin to develop a method and framework for evaluating clinical trial patient satisfaction, creating benchmarks, hearing the clinical trial patient voice, and making local improvements in response.

## Methods

### Setting

The MultiCare Institute for Research and Innovation (MIRI) is the largest medical research organization in the South Puget Sound region of Washington State. Embedded within the MultiCare community Hospital System (MHS), MIRI conducts research into more than ten therapeutic areas at 19 clinical sites. Federally funded oncology clinical trials had been conducted at MultiCare for nearly three decades prior to the launch of MIRI in its current form, and that work continues today with MIRI serving as primary recipient for the Northwest National Cancer Institute Community Oncology Research Program.

With MIRI’s establishment came a formal expansion of research endeavors into additional therapeutic areas and the development of an investigator-initiated research program. In 2014, MIRI had over 800 patients active in 87 industry-sponsored or 65 federally funded clinical trials. Fifty-three MHS employed physicians acted as principal or sub-investigators on these trials. Although MIRI has its own staff of over 20 clinical research coordinators/assistants, patients are seen in MultiCare facilities and our staff members are all MultiCare employees. Further blurring the distinction between MultiCare and MIRI, many trials include visits during which both standard-of-care and research specific procedures occur. Research patients are unlikely to make a distinction between the research and routine care aspects of their visits, thus strengthening the association between the clinical trial patient experience and patient perception of the MultiCare system as a resource for nonresearch-related medical care.

### Survey development

To create a patient satisfaction survey tailored to clinical trial patients we reviewed the literature [8, 15–18], the standardized outpatient survey distributed by a third-party vendor with which our organization contracts, surveys developed by two internal departments (Home Health and Hospice and Sleep Disorders Center), and additional patient satisfaction surveys that were freely available online. Several common areas of inquiry emerged from these reviews including satisfaction with scheduling, facility, staff/providers, overall satisfaction, and likelihood to return for further care or to recommend the facility to family/friends. We also conducted two brainstorming sessions, first as the MIRI leadership team and subsequently with our institution’s Research Oversight Committee, to identify areas of inquiry specific to the clinical trial patient experience. As the survey was to be used as part of our quality improvement process, we did not apply for Institutional Review Board approval prior to mailing it to patients. However, the MultiCare Institutional Review Board granted exemption for use of the results from this anonymous survey for manuscript preparation in April 2015 under protocol number EX-95, and return of a completed survey was considered implied consent.

Development of this survey held the dual purpose of first evaluating the feasibility of distributing and analyzing results from a clinical trial participant patient satisfaction survey, and subsequently establishing an internal baseline for use in ongoing assessment of the experience of this unique patient population until a national benchmark becomes available. The author (BMP) developed an initial draft of the survey which was then refined with input from the four-member MIRI leadership team (PJA, AR, RP, and SR) responsible for oversight of all research operations and staff. The penultimate version of the survey was then vetted by MultiCare’s Research Oversight Committee which is comprised of 18 physician leaders and high-level administrators and executives within the MultiCare system. The leadership team, in concert with the Research Oversight Committee, identified patients who had (1) consented to a trial in the past year or (2) completed a trial in the past year to which they had consented more than 1 year prior as target survey recipients. The goal of this stratification was to be able to capture a range of recent patient experiences while simultaneously identifying disparities in the perspectives of patients who were at the beginning or end of their research participation. Due to resource limitations, no follow-up phone calls or emails were made after mailing the survey.

The final instrument (see Additional file 1) included seven questions related to the study (how they learned of the study, medical condition, number of visits, etc.), 27 questions on a 5-point Likert scale (Strongly Agree, Agree, Neutral, Disagree, Strongly Disagree), two ranking questions, three free-text response options, and four demographic questions (sex, age, ethnicity, and zip code) for a total of 45 questions on three pages. The instrument also included a preface statement requesting feedback, instructing the patient to return the questionnaire in the enclosed postage-paid envelope, and informing the patient that all responses would be anonymous and kept confidential. As this was the initial foray into assessment of the clinical trial patient experience, the decision was made to allow the survey to be longer than others that we reviewed and to include several potentially related questions. We proceeded with the expectation that we would reduce the length of the survey in the following year after reflecting upon the survey administration experience, analyzing results, and identifying questions that most effectively captured the data of interest.

### Analyses

Likert scale questions were evaluated using both a “top box” score (the percentage of respondents who selected Strongly Agree for each question) and a Six Sigma percentile rank procedure [19]. The percentile rank procedure compares each question’s mean to a benchmark value of 4 (Agree) [20], incorporating the variability of the responses by normalizing the data and finding a percentile rank based on the standard normal distribution. Correlation analysis was used to evaluate associations between intended future use of our health care system for routine care and the 26 other Likert measures of patient satisfaction. Pairwise correlations between all Likert scale items were also reviewed to identify redundant questions and unexpected associations. Free-text responses were not formally analyzed, but were reviewed from a quality improvement perspective as anecdotal feedback. Surveys with missing data were excluded from analyses at the question level only. Holm-Bonferroni adjusted *p* values were calculated to account for the burden of multiple testing. All analyses were performed in the R statistical computing environment [21].

## Results

In June 2014 the survey was mailed to 226 patients identified as having (1) consented to a study in the past year (*N* = 125, 55 %) or (2) completed a trial in the past year to which they had consented more than 1 year prior (*N* = 101, 45 %). Three surveys were returned with no forwarding address and one patient died prior to receiving the survey, so the survey was believed to have been received by 222 patients (*N*_*consented*_ = 122, *N*_*completed*_ = 100). Ninety surveys (41 %) were returned between June and November 2014, 81 of which (90 %) were received within 6 weeks of the mailing. Missing data were present in six (6.6 %) surveys: four (4.4 %) were missing one data point, one (1.1 %) was missing two data points, and one (1.1 %) patient completed only the first page of the survey. A significantly higher proportion of the patients in the consented group returned the survey compared to the patients who had completed a study (50 % versus 29 %, *p* = 0.002). Forty-four respondents (48.8 %) were female, 63 (70.0 %) were aged 55 or older and 73 (81.1 %) self-identified as Caucasian. Fifty-two respondents (57.7 %) reported a home zip code within 15 miles of MIRI’s main research facility (Tables 1 and 2). Respondent distribution by therapeutic area is presented in Table 3.

**Table 1:** Demographics and background

	Survey recipients		Survey respondents		MHS patient population
	*N*	%	*N*	%	%
Female	111	49.1	44	48.9	52.4
Aged ≥55 years	135	59.7	63	70.0	24.3
Caucasian	191	85.7	73	81.1	73.6
Consented	125	55.3	61	67.8	-

*MHS* MultiCare Health System

**Table 2:** Additional respondent characteristics

	Number	Percent
Residence zip code <15 miles from main MIRI facility	59	65.6
Received care at MHS prior to study enrollment	76	84.4
Doctor recommended the study during a visit	67	74.4
Made ≥4 visits for the study	62	68.9

*MHS* MultiCare Health System, *MIRI* MultiCare Institute for Research and Innovation

**Table 3:** Survey respondent therapeutic area of study

	Survey recipients		Survey respondents		% responded within therapeutic area
Therapeutic area	*N*	%	*N*	%	%
Cardiovascular	40	17.7	18	20.0	45.0
Endocrinology	24	10.6	12	13.3	50.0
Internal medicine	3	1.3	2	2.2	66.7
Neurology	43	19.0	16	17.8	37.2
Oncology	26	11.5	10	11.1	38.5
Pediatrics	22	9.7	2	2.2	9.1
Pulmonology	62	27.4	25	27.8	40.3
Stroke	6	2.7	3	3.3	50.0
Not reported	-	-	2	2.2	-

The highest positive responses were related to patient-staff interactions, with a percentile rank of 90 or higher for staff friendliness (96.8th percentile), respect for patients (94.9th percentile), time spent with patients (94.9th percentile), explanation of role in the study (90.8th percentile), and answering questions fully (90.3th percentile; Table 4). Facility cleanliness and environment were well-rated (86.7th percentile and 81.3th percentile) but parking and convenience of location less so (68.6th percentile and 61.7th percentile). Although the rankings for satisfaction with care and the likelihood of returning to the provider were at the 83rd percentile, overall questions related to enrolling in future trials, enjoying visits, and believing that medical care was enhanced by the study ranked below the 62nd percentile. Fifty-one point one percent of patients strongly agreed that they would seek future care in our hospital system (64th percentile).

**Table 4:** Survey questions and results

Question	NA	% strongly agree	Percentile rank	Mean	*SD*	*Z* score
Friendly staff	1	80.0	96.8	4.80	0.43	1.85
Respectful staff	1	75.6	94.9	4.75	0.46	1.64
Adequate time spent	1	72.2	94.9	4.73	0.45	1.64
Staff explained role	1	66.7	90.8	4.66	0.50	1.33
Staff answered questions	1	65.6	90.3	4.65	0.50	1.30
Staff explained procedures	1	64.4	88.2	4.63	0.53	1.19
Fully informed of risks/benefits	1	63.3	87.7	4.62	0.53	1.16
Staff protected privacy	1	65.6	87.3	4.63	0.55	1.14
Trusted staff	1	64.4	86.8	4.62	0.55	1.12
Clean facility	0	66.7	86.7	4.63	0.57	1.11
Staff were prepared	1	71.1	86.5	4.66	0.60	1.10
Reasonable wait time	0	61.1	86.3	4.59	0.54	1.09
Confident in staff skills/knowledge	1	63.3	86.2	4.61	0.56	1.09
Staff responded to concerns	1	65.6	84.4	4.62	0.61	1.01
Extremely satisfied with care	1	64.4	83.3	4.60	0.62	0.97
Would return to provider for care	1	66.7	83.0	4.62	0.65	0.95
Visit times were convenient	0	61.1	81.5	4.56	0.62	0.90
Environment was pleasant	0	63.3	81.3	4.57	0.64	0.89
Scheduling visits was easy	0	63.3	78.5	4.54	0.69	0.79
Received reminders for visits	4	57.8	71.7	4.45	0.79	0.57
Parking was easy	1	52.2	68.6	4.38	0.79	0.48
Will seek future care at MHS	1	51.1	64.5	4.33	0.88	0.37
Convenient location	1	45.6	61.7	4.25	0.83	0.30
Would do another study	1	52.2	61.3	4.27	0.94	0.29
Enjoyed visits	1	38.9	59.1	4.18	0.78	0.23
Will encourage others to do studies	1	35.6	52.1	4.04	0.84	0.05
Medical care enhanced by study	1	40.0	50.0	4.00	1.00	0.00

*MHS* MultiCare Health System

*NA* represents the number of respondents who did not answer the associated question

Six items were significantly correlated with participant intention to seek future medical care at a MultiCare facility. All of these correlations were positive and moderate (0.36–0.54, Table 5), and four relate to the relationship with staff. Patient intent to return to the health care provider for routine care was most highly correlated with this item (*r* = 0.54, *p* < 0.0001). Intent to encourage others to participate in studies (*r* = 0.34, *p* = 0.0013) and enjoyment of study visits (*r* = 0.33, *p* = 0.0016), although statistically significant, had Pearson correlation values that are generally considered weak. Evaluation of all pairwise correlations showed strong correlations between perception of a pleasant environment and ease of visit scheduling (*r* = 0.85), trust in research staff (*r* = 0.77), confidence in skills/knowledge of staff (*r* = 0.72), and perception of rapid and full response to concerns (*r* = 0.70, all *p* < 0.0001).

**Table 5:** Significant correlations

Likert scale items	Correlation	*P* value	95 % CI
I will seek future medical care at a MultiCare facility:			
I would return to this health care provider for routine care	0.54	<0.001	0.38–0.67
Staff members treated me with respect	0.40	0.003	0.21–0.56
I will encourage family and friends to participate in studies	0.34	0.003	0.14–0.51
Staff responded quickly and fully to my concerns	0.38	0.005	0.19–0.55
I trusted the research staff members	0.38	0.006	0.18–0.54
Staff members were friendly	0.36	0.013	0.16–0.53
The environment was pleasant:			
Scheduling my visits was easy	0.85	< 0.001	0.78–0.90
I trusted the research staff members	0.77	< 0.001	0.67–0.85
I was confident in the skills and knowledge of the staff	0.72	< 0.001	0.61–0.81
Staff responded quickly and fully to my concerns	0.70	< 0.001	0.58–0.79

Sixty-seven respondents (74.4 %) cited contribution to medical science and potentially helping others with similar conditions as a primary motivation for volunteering for their research study. An additional 61 respondents (67.8 %) indicated that a primary motivation was the hope that their own medical condition would be improved. Financial incentives were least cited (*N* = 16, 17.8 %) as a primary motivation for participation (Table 6).

**Table 6:** Motivations for volunteering

Motivation	Number	Percent
To contribute important information to medical science	67	74.4
To potentially help other people with similar conditions	67	74.4
I hoped that the research study would improve my medical condition	61	67.8
To gain insights into my own health	42	46.7
To benefit from the additional medical attention and testing that the study provided	42	46.7
Because of the financial incentives of the study	16	17.8

## Discussion

This preliminary evaluation of research patient satisfaction demonstrates that querying this special patient population is feasible and yields valuable insight. Our response rate of 41 % is nearly 2.5 times that of the 17 % response rate to our hospital system’s vendor-based outpatient services survey. This may be reflective of a high level of engagement of patients who participate in clinical trials, or suggestive of a heightened willingness of this population to share their perspective; regardless, this response rate underscores the need for the clinical trial patient voice to be heard by the research community. Patients who had consented to a study in the past year were more likely to reply to the survey than patients who had completed a study in the past year. This could be due to a feeling of the experience being more “fresh” as study enrollment was concurrent to survey receipt. Motivations of our research patients were similar to those described in the literature [11, 12, 15, 16, 22], with altruism acting as the primary motivating factor in the decision to enroll on a trial. Our staff was highly rated by participants and free-text responses reflected satisfaction with the staff. However, in the absence of national benchmarks and systematic polling of clinical trial patients immediately following a visit it is not possible to execute a true assessment of the quality of patient-staff interactions in our research institute, as these seemingly “high” scores are not situated in a broader patient experience context and it is unknown if patients who had a negative clinical trial experience were prone to respond to this one-time mailing that may have occurred up to a year after their visit.

The observed correlations between an intent to return to our system for care and several variables were weak to moderate (0.33–0.54) and the associations warrant further explanation before basing process or organization changes on them. Fifty-four percent of the patients who indicated intent to return to MHS for routine care reported a zip code within 15 miles of our main research facility, but with system medical facilities throughout the South Puget Sound region it is difficult to ascertain the extent to which distance influenced this response. An unanticipated finding was the set of strong correlations between perception of a pleasant environment and modifiable aspects of the study, such as scheduling, trust/confidence in the staff, and satisfactory response by staff to patient concerns.

Local directions for future work include revision of the clinical trial patient survey to align with the vendor-based outpatient survey used by our institution. We believe that this will enable us to locate our own patient satisfaction profile within the broader context of patient satisfaction in our organization until national benchmarking data become available through widespread evaluation of the clinical trial patient experience. It has been shown that patient satisfaction is best assessed using surveys targeted to a specific visit rather than attempting to evaluate a general perception of an experience [6] and, moving forward, we aim to administer the survey as patient visits occur and to target evaluation of specific visits. Indeed, after reviewing responses for the survey discussed here, we revised our instrument and now distribute a two-page instrument with 27 Likert scale questions and seven demographic questions. This instrument is sent to patients immediately following a consent, annual, or end of study visit. We intend to report results from this instrument in a future publication.

A substantial limitation of this work is the lack of national benchmark data for the United States. We hope that this preliminary effort to evaluate patient satisfaction of clinical trial participants will act as a call to action in the broader research community to begin evaluation of the research patient experience and the impact of research on the patient perception and utilization of health care systems, ultimately leading to a nationally standardized system of evaluation. This is of special importance as, with any survey, the findings in this study are based on a voluntary response sample and without repeated and diverse sampling, the generalizability of the results is diminished. Such repeated sampling is also crucial if we are to identify areas of greatest importance to the patients themselves, both to improve practices nationwide and to develop more concise instruments for receiving feedback from these important volunteers. Future inquiry into research patient satisfaction would benefit greatly from a mixed-methods approach, including analysis of structured interviews, to develop a more nuanced description of the research patient experience. Ultimately, research will also be required to determine the differences in how patient satisfaction is measured and what is of greatest import for patients engaging in clinical research in countries with national health care compared to that of research patients in the United States.

We hope, with this work, to be able to share our lessons learned with other members of the research community and proceed in collaboration with other research organizations to develop and deploy a standardized survey, thereby beginning the important work of creating a foundational understanding of clinical trial patient satisfaction. Without such a system we are at risk of misunderstanding and effectively responding to the patient voice. We now have an opportunity to explore another realm of inquiry into clinical trial research and to validate the generous contributions made by patients who put themselves and their families at the forefront of medical breakthroughs.

## Conclusions

Satisfaction of patient volunteers in clinical trials is grossly understudied. In an era of increasing focus on the patient experience, this important subset of patients receiving research-related health care services needs to be queried to obtain a better understanding of how we can best serve these patients and how their experience in research impacts their use of health care systems for nonresearch-related care. Some similarities to nonresearch patient satisfaction seem to be present, such as a strong influence of interactions with care providers, but a national movement is needed among organizations conducting clinical trials in order to develop benchmarks and create and understand the connections for these patients between research-related and nonresearch-related care.

## Additional file

Additional file 1: Survey instrument.pdf (copy of instrument used to collect data reported in this manuscript). (PDF 335 kb)

## Abbreviations

HCAHPS: Hospital Consumer Assessment of Healthcare Providers and Systems
MHS: MultiCare Health System
MIRI: MultiCare Institute for Research and Innovation
