# Supplementary material for: Assessment of clinical trial participant patient satisfaction: a call to action
Source: Trials. 2016 Oct 6;17:483. doi: 10.1186/s13063-016-1616-6 (PMC5053216; doi:10.1186/s13063-016-1616-6)
Supplement: Additional file 1: — Survey instrument.pdf (copy of instrument used to collect data reported in this manuscript). (PDF 335 kb) [file 13063_2016_1616_MOESM1_ESM.pdf]

MultiCare Institute for Research & Innovation
BetterConnected

Our records indicate that you recently participated in a research study managed by the MultiCare Institute for Research & Innovation. We are conducting a patient satisfaction survey and would appreciate your feedback. Please complete this questionnaire and return it in the enclosed postage paid envelope. ***All responses are anonymous and will be kept confidential***.

BACKGROUND

1. How did you first learn about the research study?
   - ☐ My doctor recommended the study to me during a visit
   - ☐ I saw a flyer about the study
   - ☐ Someone telephoned me and invited me to participate
   - ☐ A friend or family member suggested I join the study
   - ☐ I learned about the study online
   - ☐ Other: ____

2. Please indicate which facility you visited for the majority of your study visits (check all that apply):
   - ☐ Baker Center
   - ☐ Good Samaritan Hospital Facilities
   - ☐ Tacoma General Hospital Facilities
   - ☐ Cedar Medical Center
   - ☐ Jackson Hall Medical Center
   - ☐ Other: ____________________________
   - ☐ Gig Harbor Facilities
   - ☐ Mary Bridge Children's Health Center

3. Have you completed the research study? ☐ Yes ☐ No ☐ Unsure

4. If you responded "no" to question 3, please select your reason for not completing the study:
   - ☐ I am still enrolled in the study and have not yet completed it
   - ☐ I decided I did not want to participate
   - ☐ I was told that I was not eligible for the study
   - ☐ I began the study but my participation was stopped by the study team

5. What medical condition did the study investigate (e.g. diabetes, stroke, cancer)? ___________________________________

6. Prior to this research study I received care from MultiCare Health System. ☐ Yes ☐ No

7. Please indicate the number of visits you made to the facility for the study: ☐ 1 ☐ 2 ☐ 3 ☐ 4 or more

STUDY VISITS

	Strongly Agree	Agree	Neutral	Disagree	Strongly Disagree
1. The study location was convenient.	☐	☐	☐	☐	☐
2. Parking for my visit was easy.	☐	☐	☐	☐	☐
3. Wait times for my visit were reasonable.	☐	☐	☐	☐	☐
4. The facility was clean.	☐	☐	☐	☐	☐
5. The environment was pleasant.	☐	☐	☐	☐	☐
6. Scheduling my visits was easy.	☐	☐	☐	☐	☐
7. I received reminders about upcoming visits.	☐	☐	☐	☐	☐
8. Visits were scheduled at times that were convenient for me.	☐	☐	☐	☐	☐

STUDY STAFF – Health care providers (physicians, nurses, research coordinators and research assistants)	Strongly Agree	Agree	Neutral	Disagree	Strongly Disagree
1. Staff members were friendly.	☐	☐	☐	☐	☐
2. Staff members explained their role in my care.	☐	☐	☐	☐	☐
3. Staff members explained the study procedures to me.	☐	☐	☐	☐	☐
4. I was fully informed of the risks and benefits of participation.	☐	☐	☐	☐	☐
5. Staff members answered my questions fully.	☐	☐	☐	☐	☐
6. Staff members treated me with respect.	☐	☐	☐	☐	☐
7. Staff members were well prepared for my visit.	☐	☐	☐	☐	☐
8. I was extremely satisfied with the care I received.	☐	☐	☐	☐	☐
9. I trusted the research staff members.	☐	☐	☐	☐	☐
10. I was confident in the skills and knowledge of the staff.	☐	☐	☐	☐	☐
11. Staff members protected my privacy.	☐	☐	☐	☐	☐
12. Staff responded quickly and fully to my concerns.	☐	☐	☐	☐	☐
13. Adequate time was spent with me.	☐	☐	☐	☐	☐
14. I would return to this health care provider for routine care.	☐	☐	☐	☐	☐

STUDY	Strongly Agree	Agree	Neutral	Disagree	Strongly Disagree
1. I would participate in another research study.	☐	☐	☐	☐	☐
2. I will encourage family and friends to participate in studies.	☐	☐	☐	☐	☐
3. My medical care was enhanced by participating in the study.	☐	☐	☐	☐	☐
4. I enjoyed my study visits.	☐	☐	☐	☐	☐
5. I will seek future medical care at a MultiCare facility.	☐	☐	☐	☐	☐

FUTURE PARTICIPATION

1. Please select your ***top three reasons*** for volunteering for the study.

   ☐ To contribute important information to medical science.

   ☐ To potentially help other people with similar conditions.

   ☐ I hoped that the research study would improve my medical condition.

   ☐ To gain insights into my own health.

   ☐ Because of the financial incentives of the study.

   ☐ To benefit from the additional medical attention and testing that the study provided.

   ☐ Other: ____
   ____
   ____

2. If you would choose ***not*** to participate in future research studies, please indicate the reasons why:

   ☐ There were too many blood draws or other invasive procedures.

   ☐ There were too many forms to complete.

   ☐ There were too many follow up visits.

   ☐ I did not like the study staff.

   ☐ I don't believe my privacy was sufficiently protected.

   ☐ The financial incentive was not big enough.

   ☐ Other: ____

COMMENTS

1. Was there a particular staff member(s) who made your experience positive or negative? ☐ Yes ☐ No

   If yes, please explain: ____

   ____

   ____

2. Was there any part of your experience that could have been improved? ☐ Yes ☐ No

   If yes, please explain: ____

   ____

   ____

3. If you decided to withdraw from the study, can you explain your reason?_____________________________________

   ____

   ____

DEMOGRAPHIC INFORMATION

1. Sex: ☐ Female ☐ Male

2. Age group:

☐ < 18	☐ 25-34	☐ 45-54	☐ 65-74
☐ 18-24	☐ 35-44	☐ 55-64	☐ ≥ 75

3. Ethnicity (please check all that apply):

☐ African American	☐ Asian	☐ Mediterranean	☐ Pacific Islander
☐ Alaskan Native/Eskimo	☐ Caucasian	☐ Middle Eastern	☐ Other ___________________
☐ American Indian/Native American	☐ Hispanic/Latino	☐ Multi-Racial	

4. Home zip code at the time of the study: _______________
